# Mutant AKT1-E17K is oncogenic in lung epithelial cells

**DOI:** 10.18632/oncotarget.4022

**Published:** 2015-05-25

**Authors:** Carmela De Marco, Donatella Malanga, Nicola Rinaldo, Fernanda De Vita, Marianna Scrima, Sara Lovisa, Linda Fabris, Maria Vincenza Carriero, Renato Franco, Antonia Rizzuto, Gustavo Baldassarre, Giuseppe Viglietto

**Affiliations:** ^1^ Department of Experimental and Clinical Medicine, University “Magna Graecia”, Catanzaro, Italy; ^2^ BIOGEM-Institute of Genetic Research, Ariano Irpino (AV), Italy; ^3^ Experimental Oncology 2, Centro di Riferimento Oncologico, Aviano (PN), Italy; ^4^ Experimental Oncology, IRCCS Fondazione Pascale, Napoli, Italy; ^5^ Department of Medical and Surgical Sciences, University “Magna Graecia” Medical School, Catanzaro, Italy

**Keywords:** lung cancer, AKT1-E17K, human lung epithelial cells, p27

## Abstract

The hotspot E17K mutation in the pleckstrin homology domain of AKT1 occurs in approximately 0.6–2% of human lung cancers. In this manuscript, we sought to determine whether this AKT1 variant is a *bona-fide* activating mutation and plays a role in the development of lung cancer. Here we report that in immortalized human bronchial epithelial cells (BEAS-2B cells) mutant AKT1-E17K promotes anchorage-dependent and -independent proliferation, increases the ability to migrate, invade as well as to survive and duplicate in stressful conditions, leading to the emergency of cells endowed with the capability to form aggressive tumours at high efficiency. We provide also evidence that the molecular mechanism whereby AKT1-E17K is oncogenic in lung epithelial cells involves phosphorylation and consequent cytoplasmic delocalization of the cyclin-dependent kinase (cdk) inhibitor p27. In agreement with these results, cytoplasmic p27 is preferentially observed in primary NSCLCs with activated AKT and predicts poor survival.

## INTRODUCTION

The AKT kinases (AKT1, AKT2, AKT3) represent the primary downstream end-point of the phosphoinositide 3-kinase (PI3K) pathway, regulating proliferation, survival, metabolism and invasion [1] that are frequently activated in human cancer [2], [3, 4]. Until recently aberrant AKT signalling in cancer was considered the consequence of either loss of negative regulators (PTEN) or acquisition of activating mutations/alterations of upstream regulators (i.e. tyrosine kinase receptors, RAS, p110 α-catalytic subunit of PI3K) [5, 6], [7]. In 2007 an oncogenic somatic mutation in the pleckstrin homology (PH) domain of AKT1 that results in glutamic acid to lysine substitution at residue 17 (E17K) was reported in breast, ovarian and colon cancer [8]. This change increases the binding of AKT1 to PI (4, 5) P2, enhancing plasma membrane recruitment and activation [8, 9]. Accordingly, endogenous AKT1-E17K mutant detected in lung cancer cells shows enhanced membrane localization [10], which results in the activation of downstream signalling [8, 10]. Since then, AKT1-E17K mutation has been identified at low frequency in multiple cancer types [10–17]. In lung cancer AKT1 mutations are rare, with overall reported frequency of 0.6–2% [10, 13, 18–21].

Mutant AKT1-E17K has been shown to transform murine embryo fibroblasts [8] but its role in epithelial tumorigenesis remains unclear because it apparently exerts minimal effects in immortalized breast MCF-10A epithelial cells [22] but is transforming in knocked-in breast cancer MCF-7 cells [23–25]. Similarly, no information is available for the role of AKT1-E17K mutation in lung cells so far. Lung cancer is a leading cause of cancer-related deaths, being associated with a 5-year worldwide survival rate of less than 15% [26, 27]. However, the studies reporting *PIK3CA* [28–30] and *AKT1* mutations [10, 20] in patients affected by lung cancer, and in particular in a subset with squamous cell carcinomas for which no targeted therapy is available yet, have suggested that these genes may represent relevant therapeutic targets for these patients [21, 31–33].

These considerations prompted us to address the role of AKT1-E17K in the transformation of lung epithelial cells and to investigate the molecular mechanisms involved. Herein, we demonstrate that AKT1-E17K is oncogenic in human bronchial epithelial cells and that part of its oncogenic activity is dependent upon phosphorylation and consequent cytoplasmic relocalization of the cyclin dependent kinase inhibitor p27. In addition, we provide evidence that also other alterations such as gain-of-function mutations of PIK3CA (E545K) or loss of PTEN, both of which trigger aberrant signalling through the PI3K/Akt pathway, impinge on p27 delocalization to exert their oncogenic activities.

## RESULTS

### AKT1-E17K stimulates proliferation of human lung epithelial cells

**Figure 1:**
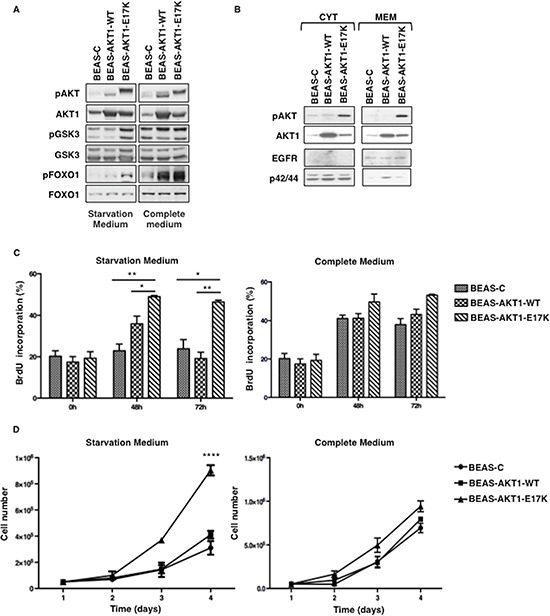
AKT1-E17K increases AKT signalling and anchorage-dependent growth **A.** Immunoblot of pAKT, AKT1, pGSK3α/β (pGSK3), GSK3α/β (GSK3), pFOXO1 and FOXO1 in BEAS-2B cells and derivatives grown in starvation or complete medium. **B.** Immunoblot analysis of pAKT, AKT1 in cytosolic- or membrane-enriched extracts. EGFR and p42/44 were used for normalization and to rule out cross-contamination. **C.** BrdU incorporation of BEAS-2B cells and derivatives grown in starvation or complete medium. The graphs show the mean ± SD. *p < 0.05, **p < 0.01. **D.** Trypan Blue exclusion assay of BEAS-2B cells and derivatives grown in starvation or complete medium. Data are means ± SD calculated using triplicate samples of a representative experiments. ****p < 0.0001.

We investigated whether mutant AKT1-E17K transforms lung epithelial cells using human normal bronchial epithelial cells immortalized by infection with Adenovirus 12/SV40 hybrid virus (BEAS-2B) [34]. This cellular system has already been used successfully to investigate the role of oncoproteins such as RAS, p53 or ERBB2 in development of lung cancer [35–37]. BEAS-2B cells were mock-transduced (BEAS-C) or transduced with lentivirus carrying wild type or mutant AKT1 (BEAS-AKT1-WT, BEAS-AKT1-E17K). BEAS-C, BEAS-AKT1-WT and BEAS-AKT1-E17K cells from different transduction experiments were expanded for further studies. The presence of the exogenous AKT1-WT or AKT1-E17K proteins in transduced cells was detected by immunoblot (Fig. 1A). BEAS-AKT1-E17K cells presented increased phosphorylation of AKT substrates such as GSK3α/β (Ser9/22) and FOXO1 (Thr256), particularly in starvation medium (Fig. 1A), increased membrane localization of mutant AKT1 compared with endogenous or transfected wild type protein as well as increased pS473 phosphorylation of mutant AKT1 (Fig. 1B).

Analysis of cell proliferation by measure of BrdU incorporation demonstrated that BEAS-C, BEAS-AKT1-WT and BEAS-AKT1-E17K cells carry out DNA synthesis efficiently in complete medium (BrdU incorporation rate at 48 hours from plating was of 41.1 ± 1.7%, 41.2 ± 2.5% and 49.6 ± 4.1%, respectively) (Fig. 1C). Conversely, when grown in the absence of growth factor BEAS-C cells showed significantly decreased rate of BrdU incorporation (22.9 ± 3.2% and 23.8 ± 4.4% at 48 and 72 hours from plating, respectively), whereas BEAS-AKT1-E17K cells continued to carry out DNA synthesis at high efficiency (48.9 ± 0.6% and 46.4 ± 0.9% at 48 and 72 hours from plating, respectively). BEAS-AKT1-WT cells showed an intermediate behaviour (Fig. 1C). Accordingly, BEAS-AKT1-E17K cells duplicated at an accelerated rate in monolayer compared with BEAS-C cells or with the corresponding cells transduced with wild type AKT1 (Fig. 1D). In agreement with the results of AKT activation, the difference in the proliferation rate between BEAS-AKT1-E17K cells and BEAS-C or BEAS-AKT1-WT cells was predominantly observed under conditions of growth factor deprivation.

These results indicate that mutant AKT1-E17K, but not wild type AKT1 expressed at similar level, is able to promote anchorage-dependent proliferation of human bronchial epithelial cells, especially under stressful conditions.

### AKT1-E17K stimulates migration and invasion of human lung epithelial cells

**Figure 2:**
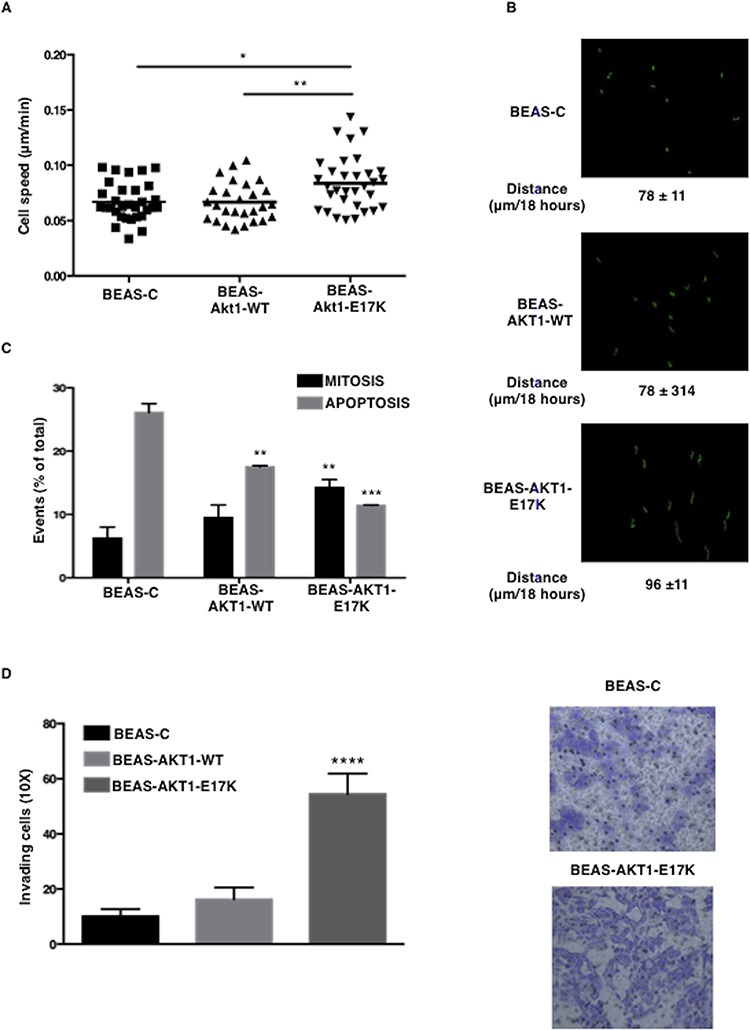
AKT1-E17K promotes motility **A.** Speed (μm/min) of indicated cell lines included in 3D Matrigel. *p < 0.05, **p < 0.01. **B.** Cell tracking analysis of cells included in 3D Matrigel. In green the trajectories depicted by indicated cell lines. The mean path length is reported for each cell line. **C.** Percentage of dividing (black bars) or dead (gray bars) cells included in 3D collagen I in the absence of serum for 24 hours. **p < 0.01, ***p < 0.001. **D.** Invasion assay in modified Boyden chambers. Left: median number of invading cells/field. ****p < 0.0001. Right: representative images of invading cells (Magnification 20X).

We also evaluated the effects of AKT1-E17K on the capability of bronchial epithelial cells to migrate. All cells were included in 3D Matrigel matrices and followed for 18 hours using time-lapse microscopy [38]. No difference in cell motility was observed in the presence of growth factors (0.09 μm/min) (not shown). In 3D setting BEAS-C cells moved with a reduced mean speed of 0.06 ± 0.01 μm/min demonstrating low velocity in serum free conditions. Expression of AKT1-WT did not significantly modify 3D velocity of BEAS-2B cells (0.06 ± 0.003 μm/min *p* = 0.8). Conversely, AKT1-E17K significantly increased the ability of the cells to move (0.1 ± 0.01 μm/min *p* = 0.01 vs control parental cells; *p* = 0.006 vs AKT1-WT expressing cells) (Fig. 2A). The total path described by each cell was significantly longer in AKT1-E17K-expressing cells respect to control or AKT1-WT expressing cells, demonstrating that AKT1-E17K prevents the reduction of cell speed due to serum deprivation (Fig. 2B).

Subsequently, we determined whether mutant AKT1 also affected cell viability under stress conditions. BEAS-2B cells and derivatives were included in 3D collagen I and the capability to divide and/or survive was analysed using time-lapse microscopy over a period of 24 hours (Supplemental Videos 1 and 2). Data reported in Fig. 2C demonstrated that only 7% of control BEAS-C cells underwent divisions while more than 25% died; cells expressing AKT1-WT showed similar rate of mitotic division (9% *p* = not significant) and a moderate decrease in the amount of dead cells (17.5% of apoptosis; *p* = 0.005 vs BEAS-C). Conversely, AKT1-E17K significantly improved survival (11.5% of apoptotic cells; *p* < 0.001 vs both BEAS-C and BEAS-AKT1-WT cells, respectively) and the ability to complete a mitotic division over the same period of time (14% of completed division; *p* = 0.005 vs both BEAS-C and BEAS-AKT1-WT cells, respectively). Finally, we evaluated the invasive potential of AKT1-E17K cells using Boyden chambers (Fig. 2D). The number of BEAS-AKT1-E17K cells that crossed the Matrigel barrier after 48 h was ~2-fold higher than that of BEAS-C or BEAS-AKT1-WT cells in medium deprived of growth factors, demonstrating that mutant AKT1-E17K is able to promote invasion through the basement membrane, a key event during tumour dissemination. These results indicate that mutant AKT1-E17K, but not wild type AKT1, is able to promote survival and mitosis in stressful conditions, and increase pro-migratory and pro-invasive potential of human bronchial epithelial cells.

### AKT1-E17K increases tumorigenicity and metastatic potential of human lung epithelial cells

**Figure 3:**
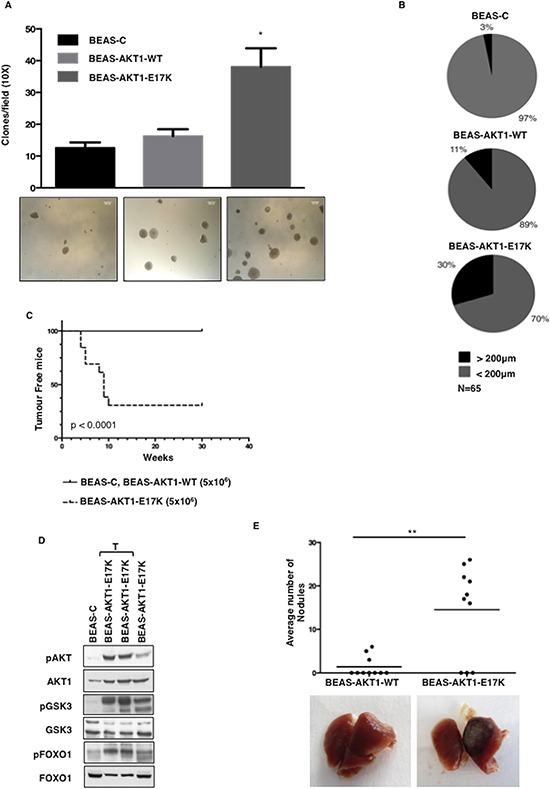
AKT1-E17K increases anchorage-independent growth and tumour formation **A.** Soft agar colony formation assay. Bars represent the mean of a representative triplicate experiment ± SD. *p < 0.05. Representative images of colonies are shown in the panels below. **B.** Average clone size was quantified using an image analysis software (ImageJ). N denotes the number of total clones scored. **C.** Kaplan-Meier curve showing tumour appearance in athymic CD1 mice (13 mice/group). **D.** Immunoblot analysis of pAKT, AKT1, pGSK3α/β, GSK3α/β, pFOXO1 and FOXO1 in tumours generated by BEAS-AKT1-E17K cells. Lysates from BEAS-C (first lane) and BEAS-AKT1-E17K cells (fourth lane) were used as negative and positive controls, respectively. T: lysates from explanted tumours. **E.** Tail vein injection assay for BEAS-AKT1-WT and BEAS-AKT1-E17K cells. Upper panel: dispersion graph showing the average number of nodules/lung/mouse; lower panel: representative images of explanted lungs. **p < 0.01.

Mutant AKT1 also stimulated anchorage-independent growth *in vitro* and tumorigenicity *in vivo*. In semisolid medium BEAS-C cells give rise to some scattered small colonies whereas AKT1-E17K, but not wild type AKT1, induced a 3-fold increase in the number of colonies (BEAS-C: 12.6 ± 1.7 colonies/field; BEAS-AKT1-WT: 16.3 ± 2.1 colonies/field; BEAS-AKT1-E17K: 38.1 ± 5.8 colonies/field) (Fig. 3A). Moreover, the colonies formed by BEAS-AKT1-E17K were much larger than that generated by BEAS-C or BEAS-AKT1-WT cells (Fig. 3B), a characteristic regarded as a hallmark of malignant cells [39].

In addition, when injected subcutaneously into athymic CD1 mice (5 × 10^6^ cells per mouse), mutant AKT1-E17K was able to promote tumour formation in 9 out of 13 mice with a latency of 4–10 weeks as illustrated in the Kaplan-Meier curve of Figure 3C while BEAS-C and BEAS-AKT1-WT cells were unable to grow *in vivo* (*n* = 13). Tumours were classified as poorly differentiated squamous cell carcinoma in 4 cases and poorly differentiated adenocarcinoma in 5 cases (data not shown) with marked activation of AKT signalling as determined by immunoblot for pAKT (S473) and pGSK3α/β (9/21) (Figure 3D). Finally, we observed that mutant AKT1 significantly increased the capability of human pulmonary cells to colonize lungs in mice after tail vein injection (Fig. 3E).

These results were very similar to those obtained in a cellular model of NSCLC with low activity of endogenous AKT (NCI-H23) (see Supplemental Results and Fig. S1 and S2), which indicate that the mutant AKT1-E17K allele is oncogenic for human lung epithelial cells *in vitro*.

### Mutant AKT1-E17K promotes proliferation, migration and invasion of lung epithelial cells through the delocalization of cyclin-dependent kinase inhibitor p27

The most prominent effect exerted by AKT1-E17K in human lung epithelial cells is the stimulation of proliferation and migration. AKT exerts its oncogenic effects by phosphorylating and thereby regulating the intracellular levels and/or localization of a variety of downstream substrates [40–42]. One of such substrates is the cyclin-dependent kinase inhibitor p27 [43–46]. The best understood function of p27 is the regulation of cell cycle progression through the inhibition of cdk-dependent phosphorylation of nuclear retinoblastoma protein [47]. Previous work from our lab has shown that activated AKT induces phosphorylation-dependent nucleus-to-cytoplasm shuttling of p27 [43–46, 48], which results in the impairment of its anti-proliferative activity [49–51]. Moreover, recent studies have involved cytoplasmic p27 in the regulation of cell migration, raising the possibility that, when in the cytoplasm, p27 may have additional, proliferation-independent functions that may even favour tumor development [52, 53].

**Figure 4:**
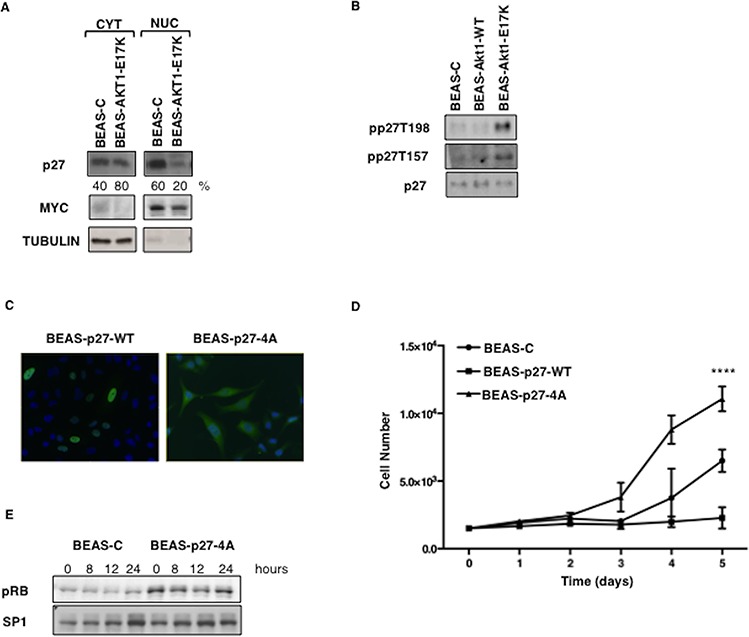
Mutant AKT1-E17K promotes cytoplasmic delocalization of p27 **A.** Immunoblot analysis of p27 localization in BEAS-2B cells and derivatives. MYC and Tubulin were used as loading controls and to rule out cross-contamination. **B.** Immunoblot analysis of phospho-T197/198, phospho-T157 and p27 in BEAS-2B cells and derivatives. **C.** Localization of exogenously expressed p27-WT and p27-4A in BEAS-2B cells by immunofluorescence. **D.** Trypan Blue exclusion assay in BEAS-2B cells transfected with control pcDNA3, p27-WT and p27-4A. Data are means ± SD of triplicate samples of a representative experiment. ****p < 0.0001. **E.** Immunoblot analysis of phosphorylated pRB. BEAS-2B control cells and BEAS-p274A were starved for 48 h and then re-stimulated with complete medium for the indicated times. SP1 was used as loading control.

Thus, we investigated whether the oncogenic effects elicited by AKT1-E17K were dependent on the phosphorylation and delocalization of p27. Immunoblot analysis showed that BEAS-2B cells expressing AKT1-E17K showed markedly decreased amount of nuclear p27 compared with control BEAS-C cells (Fig. 4A; 20% versus 60%), and higher levels of p27 phosphorylation at T157 and 198, respectively (Fig. 4B).

Similarly, compared to the corresponding BEAS-C cells, BEAS cells expressing mutant PIK3CA or silenced for PTEN showed a decreased amount of nuclear p27 compared with control BEAS-C cells (Supplemental Fig. S3A; 31% in BEAS-PI3K-E545K (BEAS-PIK3CA), 43% in BEAS-shPTEN versus 58% of Control), and higher levels of p27 phosphorylation at T157 and 198, respectively (Supplemental Fig. S3B). Altogether, these results demonstrated that aberrant signalling through the PI3K pathway – induced by mutant AKT1, mutant PIK3CA or by PTEN loss – is associated with T157/T198 phosphorylated p27, which is delocalized in lung epithelial cells.

To obtain formal evidence that AKT-dependent relocalization of p27 was able to dysregulate cell proliferation and migration/invasion of lung epithelial cells, we made use of a mutant form of p27 in which the four residues that are known to be responsible for p27 localization (K153, R154, K165, R166) [54] were replaced with alanine by site-directed mutagenesis (p27-4A) (Fig. 4C). Cytoplasmic p27-4A was able to increase cell proliferation in BEAS-2B cells compared with empty vector-transfected cells (BEAS-C); conversely wild type p27 completely blocked proliferation of BEAS-2B cells, indicating that these cells were still sensitive to inhibition of cell cycle progression by nuclear p27 (Fig. 4D). Accordingly, BEAS cells expressing p27-4A, showed increased levels of pRB phosphorylation, indicating reduced inhibition of cdks (Fig. 4E).

**Figure 5:**
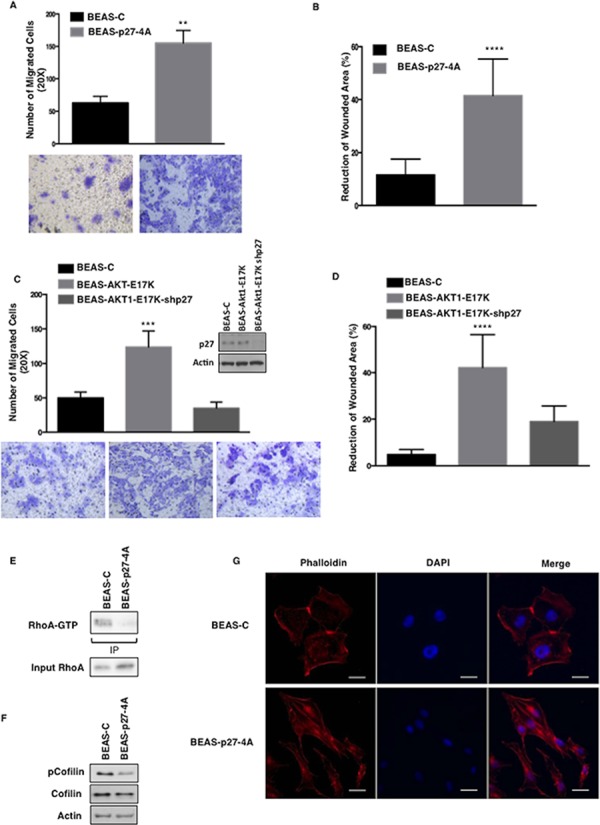
Mutant AKT1-E17K increases cell motility through cytoplasmic delocalization of p27 **A.** Migration assay of BEAS-C and BEAS-p274A cells in Boyden chamber. The graph represents the mean number (± SD) of migrated cells/field. Bottom panels, representative images of migrated cells (magnification 20X). **p < 0.01. **B.** Wound healing assay of BEAS-C and BEAS-p274A cells. The graph represents the percentage of wound reduction after 48 h. ****p < 0.0001. **C.** Boyden chamber migration assay of BEAS-C, BEAS-AKT1-E17K and BEAS-AKT1-E17K-shp27. The graph represents the mean number (± SD) of migrated cells/field; bottom panels: representative images of migrated cells (magnification 20X). ***p < 0.001. In the inset western blot analysis of p27 in BEAS-C, BEAS-AKT1-E17K and BEAS-AKT1-E17K-shp27 cells. **D.** Wound healing assay of BEAS-C, BEAS-AKT1-E17K and BEAS-AKT1-E17K-shp27. The graph represents the percentage of wound reduction after 48 h.****p < 0.0001. **E.** Immunoblot analysis of BEAS-C and BEAS-p274A cells: immunoprecipitated GTP-bound RhoA, total RhoA. **F.** Immunoblot analysis of BEAS-C and BEAS-p274A cells: phosphorylated and total cofilin. **G.** Cytoskeletal organization of BEAS-C and BEAS-p274A cells. F-actin stained red with rhodamine-phalloidin and nuclei stained blue with DAPI. Scale bar: 20 μm. Original magnifications: 400x.

On the other hand, when BEAS-C and BEAS-p27-4A were analysed for their migratory capabilities in Boyden chamber assays and in wound healing assays, we found that whereas wild type p27 had little effect on the migration of BEAS cells (not shown), cytoplasmic p27 increased migration of BEAS-2B cells of approximately 2.5-fold in chamber assays (Fig. 5A) and increased the reduction of wounded areas by a factor of 3.6-fold (Fig. 5B). See Supplemental Fig. S4A for representative images. These results were complemented by knocking-down p27 with specific shRNAs. Suppression of p27 expression in BEAS-AKT1-E17K significantly reduced the number of migrated cells (35.3 ± 8.6 vs 123.9 ± 23.2, respectively) in Boyden chamber assays (Fig. 5C) and in wound healing assays (Fig. 5D). See Supplemental Fig. S4B for representative images of wound closure. Notably, in BEAS-2B cells, cytoplasmic p27 induced a reduction in the levels of cofilin phosphorylation, a biochemical marker of Rho kinase activity, suggesting that cytoplasmic p27 inhibited RhoA activity (Fig. 5E–5F).

In agreement with the concept that changes in actin cytoskeleton represent a hallmark of migrating cells [55, 56], cytoplasmic p27 induced BEAS-2B cells to assume more elongated morphology with reduced cell-cell contacts and the appearance of condensed actin-rich structures aligned with the long axis. Conversely, BEAS-2B control cells displayed a large, flattened morphology with a random orientation of actin filaments that appear closely adherent along their lateral and apical surfaces as detected by phalloidin staining (Fig. 5G).

**Figure 6:**
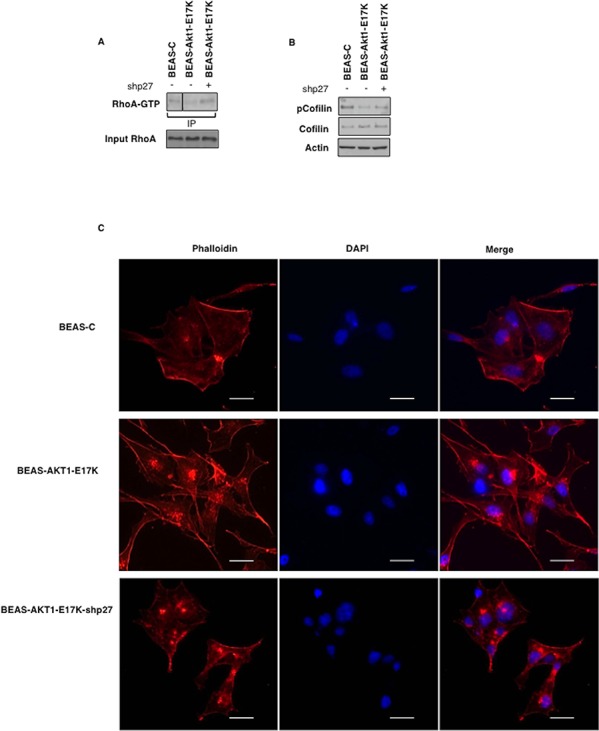
p27 is required for motility induced by mutant AKT1-E17K **A.** Upper panel: immunoprecipitated GTP-bound RhoA from indicated cell lines; lower panel: immunoblot analysis of RhoA levels in the corresponding lysates. **B.** Immunoblot analysis of phosphorylated and total cofilin in BEAS-2B and derivatives. **C.** Cytoskeletal organization of BEAS-C, BEAS-AKT1-E17K and BEAS-AKT1-E17K-shp27 cells F-actin stained red with rhodamine-phalloidin and nuclei stained blue with DAPI. Scale bar: 20 μm. Original magnifications: 400x.

Accordingly, BEAS-AKT1-E17K cells presented a cytoskeletal organization similar to that observed in BEAS-p274A cells. BEAS-AKT1-E17K cells acquired an elongated morphology with formation of actin-rich structures mainly localized in the lamellipodia and presented reduced amount of activated Rho (Rho-GTP) as well as reduced cofilin phosphorylation (Fig. 6A–6C). On the contrary, knock-down of p27 in BEAS-AKT1-E17K cells (BEAS-AKT1-E17K-shp27 cells) restored Rho-GTP levels and cofilin phosphorylation (Fig. 6A–6B) and conferred a flattened morphology with actin filaments almost localized adherent to their lateral and apical surfaces (Fig. 6C).

Altogether these results demonstrate that relocalization of p27 to the cytoplasmic compartment mediates, at least in part, the proliferative and the migratory effects induced by AKT1 in lung epithelial cells

### Activation of PI3K/AKT pathway in human NSCLC is associated with cytoplasmic localization of p27 that predicts poor clinical outcome

Subsequently, we investigated the relevance of PI3K-dependent p27 delocalization in the clinical setting of NSCLC patients, by analysing the correlation between AKT activity and p27 in samples resected from 110 NSCLC patients and arrayed onto Tissue Micro Arrays (TMAs LC1.0 and LC2.0, respectively). The analysis performed here included samples that presented mutations in AKT1, PIK3CA and KRAS, polysomy or gene amplification in AKT1 and PIK3CA and loss of PTEN expression as described previously [10, 57].

As a read-out of AKT activity in NSCLC we determined the phosphorylation status of residue S473 of AKT1 (pAKT) on TMAs containing duplicated core biopsies of NSCLC samples. The evaluation criteria used for pAKT1 staining, p27 staining and p27 localization are described in Materials and Methods. The number of tumors for which pAKT and p27 were simultaneously available was 83. The results obtained from pAKT and p27 staining in NSCLC are summarized in Tables S1 and S2. We observed AKT activation in 37 out of 83 NSCLC analysed (45%). Conversely, p27 expression was lost in 15/83 NSCLC analysed (18%). As for p27 localization, we found that p27 was high nuclear/low cytoplasmic in 17 cases, high cytoplasmic in 51 cases (Table 1). Representative patterns of p27 expression and p27 localization in NSCLC are shown in Supplemental Figure S5.

**Table 1:** Correlation between pAKT staining and p27 expression and localization in NSCLC

p27					
		Nuclear	Cytoplasmic	N^a^	*P value*
pAKT	**Negative**	13	22	35	0.02
	**Positive**	4	29	33	
	**N**^a^	17	51	68	

a Patients for which both p27 and pAKT staining were available

**Figure 7:**
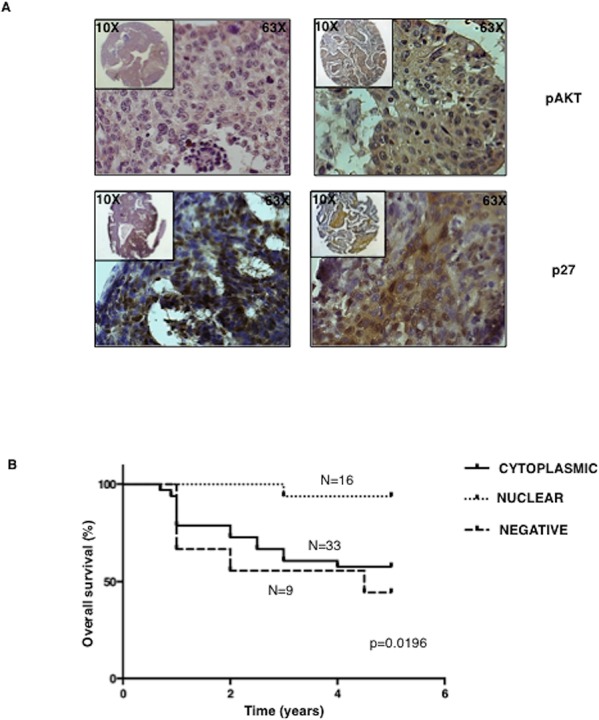
Cytoplasmic p27 localization correlates with activated AKT and predicts poor prognosis in NSCLC patients **A.** Left, representative immunostaining of a NSCLC sample scored negative for AKT activation (upper panel) with nuclear p27 localization (lower panel). Right, representative immunostaining of a NSCLC sample scored positive for AKT activation (upper panel) and showing cytoplasmic p27 localization (lower panel). Magnification as indicated. **B.** Kaplan-Meier curve of NSCLC patients with high, reduced or mislocalized p27 protein.

We did not find any correlation between AKT activation and p27 levels: 33 out 37 tumours with active AKT were at the same time positive for p27 expression (Table S2). Conversely, we found a significant correlation between active AKT and the presence of p27 in the cytoplasmic compartment. Tumors with active AKT presented cytoplasmic p27 in 29/33 cases at difference with pAKT-negative tumors (Table 1). Representative pAKT and p27 immunostainings are shown in Figure 7A. These findings suggested that cytoplasmic relocalization of p27 is the result of AKT activation induced by multiple genetic alterations typical of NSCLC that include mutations of KRAS, PIK3CA or AKT1, amplification of AKT1 or PIK3CA and/or loss of PTEN.

Notably, AKT-dependent p27 delocalization had an important impact on survival of NSCLC patients, as shown in the Kaplan-Meier curves of Figure 7B. In fact, the presence of cytoplasmic mislocalization was associated with a poorer prognosis [total average overall survival: 3.9 years (*n* = 58); average overall survival of patients with loss of p27 staining: 3.3 years (*n* = 9); average overall survival of patients with nuclear p27 staining: 4.9 year (*n* = 16); average overall survival of patients with cytoplasmic p27 staining: 3.6 years (*n* = 33)].

Altogether these results demonstrate that relocalization of p27 into the cytoplasmic compartment is a relevant aspect of aberrant AKT signalling in NSCLC, predicting poor survival in human NSCLC patients.

## DISCUSSION

The hotspot E17K mutation in the PH domain of AKT1 occurs in approximately 0.6–2% of human NSCLC patients. However, its role in the development of lung cancer remains to be explained. In this manuscript, we have investigated whether and how mutant AKT1 is oncogenic for lung epithelial cells. To this aim we have genetically engineered immortalized human bronchial epithelial cells with mutant AKT1-E17K. The most prominent findings reported in this manuscript are that: i) AKT1-E17K stimulates cell proliferation, migration and invasion of human lung epithelial cells; ii) AKT1-E17K cooperates with pre-existing alterations (i.e. loss of p53 and/or pRb in BEAS-2B cells; unknown alterations in NCI-H23 cells) to induce full malignancy in human lung epithelial cells; iii) the mitogenic and migratory effects exerted by mutant AKT1-E17K are mediated by the cytoplasmic delocalization of the cyclin dependent kinase inhibitor p27; iv) cytoplasmic p27 induced by activated AKT predicts poor survival in human NSCLC patients. These results are commented more extensively below.

In cellular systems, mutant AKT1-E17K promotes anchorage-dependent and -independent proliferation, increases the ability to migrate and invade through reconstituted basal membrane as well as to survive and duplicate in stress conditions *in vitro*, leading to the emergence of a cell population endowed with the capability to form aggressive, undifferentiated tumours at high efficiency that are able to disseminate and colonize lungs in mice. In this sense, it appears that mutant AKT1-E17K behaves in a manner similar to other potent oncogenes like RAS [34, 35] and mutant p53 [36] that significantly contribute to the pathogenesis of lung cancer. In contrast to the results reported herein, the role of AKT1-E17K in mammary cells is more controversial, since it produced no substantial effects in the non-tumorigenic mammary epithelial cell line MCF-10 [22], whereas it is apparently transforming in breast cancer MCF-7 cells [23, 24], a discrepancy likely due to intrinsic differences in the genetic constitution of the cell of origin. Accordingly, a recent study showed that AKT1-E17K inhibits growth, migration, and protein synthesis in myoepithelial breast cells whereas it enhances cell survival and migration in luminal cells [25] possibly offering an explanation to the finding that the E17K mutation occurs in breast cancer of luminal origin [58].

AKT lies at the center of signalling pathways controlling cell survival and cell death, and AKT activation is associated with resistance to apoptosis, as well as increased survival, growth, migration and angiogenesis [59]. Each of these functions is mediated by phosphorylation of specific substrates that usually results in change of stability, activity and/or localization of the implicated protein [40–42, 60]. In particular, data from different groups worldwide have contributed to identify the cyclin-dependent kinase inhibitor p27 as a critical substrate of AKT1 activity. AKT1 phosphorylates p27 at residues T157 and T198 excluding it from the nucleus and impairing its ability to inhibit cell cycle progression [43–46, 61].

Thus we asked whether the transformation of bronchial epithelial cells by AKT1 is a direct consequence of p27 deregulation. Several findings of this manuscript indicate that mutant AKT1 phosphorylates p27, induces its cytoplasmic localization and blocks p27-dependent inhibition of pRB phosphorylation by cyclin/cdks. BEAS-2B cells expressing mutant AKT1-E17K present increased phosphorylation (T157/T198) and impaired localization of p27. These cells present a marked growth advantage compared with parental cells, similarly to BEAS-2B cells expressing cytoplasmic p27 (BEAS-p27-4A). These results are in agreement with recent studies showing that urethane-induced lung tumors showed reduced nuclear/cytoplasmic ratio of p27 protein and increased T198-phosphorylated p27 in the cytoplasmic pool, and that AKT inhibition in murine lung tumour cell lines and in tumour-bearing mice led to a reduction in p27 T198 phosphorylation and its redistribution to the nucleus [62].

It is of note that also other upstream alterations that activate AKT signalling such as the loss of PTEN phosphatase or the mutations of the catalytic subunit of PI3K increase p27 phosphorylation at T157/T198 and impair its localization, thus suggesting that inactivation of p27 by phosphorylation represent a general mechanism whereby abnormallly active PI3K/AKT pathway signals.

The results presented here demonstrate also that impairment of p27 localization is part of the mechanism whereby mutant AKT1 stimulates migration and invasion in lung epithelial cells. Cytoplasmic p27 binds to the small GTPase RhoA reducing the level of RhoA-GTP, which results in the inhibition of ROCK1 kinase (a major downstream effector of RhoA-GTP). ROCK1 inhibition causes an increase in cofilin actin-depolymerization activity by reducing cofillin phosphorylation thus resulting in increased migration. In agreement with this model, we show that, on one hand, AKT1-E17K stimulates migration and invasion whereas interference with p27 expression with shRNA abolishes the promigratory/invasive effects exerted by mutant AKT1; on the other hand, BEAS-2B cells expressing cytoplasmic p27 present marked increased migratory/invasive properties that are associated with increased levels of RhoA-GTP and decreased phosphorylated cofilin. These conclusions are in agreement with previous results showing that phosphorylation of T198 by AKT1 not only mislocalizes p27 to the cytoplasm but also promotes RhoA-p27 interaction and RhoA pathway inhibition, thus stimulating cell motility [63–65].

A further confirmation of the importance of p27 delocalization induced by activated AKT1 observed in cultured cells *in vitro* comes from immunostaining experiments carried out in a cohort of human NSCLC patients showing AKT activation. Analysis of NSCLC patients indicated that tumors with high AKT activity presented significant levels of p27 in the cytoplasmic compartment, suggesting that the phosphorylation of T157/T198 residues impairs nuclear import and/or increase nuclear export. These results nicely complement the recent finding that treatment of mice bearing lung tumors with the PI3K inhibitor LY294002 induced a rapid decrease in phosphorylated AKT and phosphorylated p27, concomitant with an increase in nuclear p27 levels [62]. Notably, the presence of p27 in the cytoplasmic compartment predicts poor survival for NSCLC patients under study, indicating that AKT-dependent cytoplasmic p27 relocalization has an important clinical significance.

In summary, the conclusion drawn by the experiments reported here is that mutant AKT1-E17K is an oncogene that can transform human immortalized lung epithelial cells and that the relocalization of the cyclin-dependent kinase inhibitor p27 is a key mediator of the oncogenic activity exerted by the E17K mutation of AKT1, with cytoplasmic relocalization of p27 being predictive of poorer prognosis in NSCLC patients.

## MATERIALS AND METHODS

### Cell culture and transfections

BEAS-2B cells were purchased from ATCC-LGC Standards (Milan, Italy) and cultured in bronchial epithelial cell growth medium (BEBM) supplemented with growth factors (BEGM) (Cambrex Bio-science, Walkersville, MD) (Complete Medium). Cells were kept in culture for less than six months. Experiments were performed using cells with less than 10 culture passages. Starvation was carried out in BEBM supplemented with 0.2% BSA (Starvation Medium).

### Virus generation and Infection

Human AKT1 cDNA was purchased from Addgene (Plasmid 9021) [66]. The E17K mutation was introduced with the QuickChange Site-Directed Mutagenesis Kit (Stratagene Cloning Systems, North Torrey Pines Road, La Jolla) and verified by DNA sequencing. cDNAs were cloned in pENTR1A vector (Invitrogen, Carlsbad, CA) and recombined in pLenti-DEST-6.2 by Gateway Technology (Invitrogen). Generation of lentivirus was performed as described [67]. Transduced cells were selected with 5 μg/ml blasticydin (Invitrogen).

### Western blot and antibodies

Whole cell extracts were prepared with NP-40 lysis buffer (10 mM Tris–HCl pH 7.5, 150 mM NaCl, 1% NP-40) containing protease inhibitors (SigmaFast, Sigma-Aldrich). Western blot analysis were carried out by standard methods with the exception of experiments reported in Fig. 5E and Supplemental Fig. S3 where chemioluminescence was detected with Alliance Mini WL2M system (Uvitec, Cambridge, UK). Membrane and cytosol enriched extracts were prepared with FractioPrep Cell Fractionation kit (BioVision, Mountain View, CA). Densitometric analysis of gel bands was carried out with ImageJ software (NIH, Bethesda, MD): the sum of band intensities in the cytoplasm and nuclear/membrane compartments was set as 100%. Anti-phospho-AKT (Ser473) (#4058), anti-AKT1 (#2938), anti-p42/44 (#9107), were purchased from Cell Signaling Technology (Danver, MA); anti-phospho-p27 (Thr157) (AF1555) and anti-phospho-p27 (Thr197/8) (AF3994) were from R&D Systems (Minneapolis, MN); anti-p27 (sc-528), anti-MYC (sc-40), anti-tubulin (sc-8035), anti-phospho-cofilin (sc-12912), anti-cofilin (sc-33779) were from Santa Cruz Biotechnology (Santa Cruz, CA); anti-RhoA was from Millipore (Billerica, MA); anti-β-actin (clone AC-74, #A2228) was from Sigma-Aldrich.

### *In vitro* proliferation assay

Cells were plated at a density of 5 × 10^4^/well in triplicate, harvested, diluted 1:1 with 0.4% Trypan Blue solution (Sigma-Aldrich) and daily counted. Experiments were repeated at least three times.

### 5-Bromo-2′-deoxy-uridine (BrdU) incorporation assay

Cells were plated onto coverslips in 6-well culture plates in complete BEBM and cultured with or without growth factors for 48 and 72 h. BrdU was added 60 min prior to processing. Detection of BrdU incorporation was carried out according to manufacturer's instructions (5-Bromo-2′-deoxy-uridine Labeling and Detection Kit I, Roche Diagnostics) and visualized by microscopy (Axioplan 2, Zeiss). BrdU incorporation was measured by counting positive nuclei in a total of 500 cells in triplicate experiments.

### Time-lapse microscopy

Time-lapse microscopy and cell tracking analyses were performed as previously described [38, 68]. Cells were detached and suspended in buffered Collagen I solution, pH 7.4, at 1.67 mg/ml of final concentration (Vitrogen) or Matrigel™ (6 mg/ml, BD Biosciences) and then overlaid with serum-free medium or complete medium. Pictures/images were collected every 5 minutes for 18 hours with a Leica Time Lapse AF6000LX workstation. Collected images were used to create a movie (10 images/second) and analyzed with a cell tracking software (Leica MMAF110): 20/field cells were randomly selected and their x/y coordinates tracked using the IM2000 software to obtain cell speed (μ/min) and total distance covered (μ). At least 3 independent wells/cell types have been analysed.

### Matrigel invasion assay

Cells (1 × 10^5^) were seeded in the upper chamber of the Transwell inserts onto polycarbonate filters coated with 100μl of Matrigel (BD) and incubated for 48 h at 37°C in growth factor-deprived medium. Cells that had migrated to the bottom of membranes were fixed in cold methanol for 10 min and stained with 0.01% crystal violet in 20% ethanol. Migrated cells were counted in 5 fields/well in triplicate experiments.

### Soft agar assay

Cells (2.5 × 10^3^) were suspended in BEBM, containing 0.35% low-melting agarose (Type VII, Sigma-Aldrich) and seeded onto 0.5% low-melting agarose in six-well tissue culture plates. Colonies were scored after 3 weeks in 5 randomly selected fields/well, at 4X magnification. The experiments were repeated at least three times.

### Tumor formation assays

Cells harvested from culture plates were resuspended in 200 μl of 1:1 Matrigel in PBS and injected subcutaneously into 6 week-old athymic CD1 mice (Charles River Laboratories International, Inc., Wilmington, MA). Tumour size was calculated as length × width × width/2 with a caliper every 7 days.

For tumor dissemination experiments, 5 × 10^5^ cells were injected into the tail veins of athymic nude mice (ten mice per group). Four weeks after the injection mice were sacrificed. Tumor metastasis was determined by counting the number of nodules in each lung under a dissecting microscope.

### Wound healing and migration assay

For wound healing assay cells were plated at equal density in duplicate six-well plates and grown to confluence. Wounds were then generated with a sterile pipette, cells were rinsed twice with PBS, and fresh culture medium was added. Areas of wound were marked and photographed at 0 and 48 h (Nikon Eclipse TE 2000-S). Wound area was measured with ImageJ software. Five different wound areas/well were analysed. Transwell migration assay was performed using six-well Transwell polycarbonate filters (Sigma-Aldrich) with 8-μm pore size. Cells (5 × 10^4^) were seeded in the upper chamber of the Transwell insert and incubated for 48 h at 37°C in serum-free medium. Cells that did not migrate through the pores were manually removed with a cotton swab. Cells that migrated to the bottom of the membrane were fixed in cold methanol for 10 min and then stained with 0.01% crystal violet in 20% ethanol. Migrated cells were counted in 5 fields/well in triplicate experiments.

### Rho-GTP pull-down assays

GTP-bound Rho was assayed with Rho assay reagent (Millipore) by lysis in Rho buffer (25 mM Hepes, pH 7.5, 150 mM NaCl, 1% Igepal CA630, 10 mM MgCl2, 1 mM EDTA, 10% glycerol) plus protease inhibitors. Five hundred μg of cleared lysate were incubated with 25 μg of Rho assay slurry containing Rhotekin RBD agarose or GST-bound Sepharose, at 4°C for 1 h. Beads were washed 3 times in Rho buffer and suspended in 2x Laemmli buffer. GTP-bound RhoA was identified by immunoblot.

### Analysis of cytoskeleton

To analyze cytoskeletal organization, cells grown on glass slides to semi-confluence, were fixed with 2.5% formaldehyde, permeabilized with 0.1% Triton X-100 for 10 minutes at 4°C, and then incubated with 0.1 μg/ml rhodamine-conjugated phalloidin (Sigma-Aldrich) for 40 minutes. After nuclear staining with 4-6-diamidino-2-phenylindole dye (DAPI), cells were analysed by a fluorescence inverted microscope connected to a videocamera (Carl Zeiss).

### Patients

Archive material from 110 patients diagnosed of NSCLC was obtained from INT “Fondazione Pascale” (Naples, Italy). Patient accrual was conducted according to internal Review Board of the INT Fondazione Pascale (Naples, Italy) (CEI 556/10 of 12/3/2010). The study was approved by the internal Review Board of the AOU Mater Domini/University Magna Graecia (Catanzaro, Italy) in the meeting of 16/3/2011. Median age of patients was 63 year old (range 28–79). Among patients with clinical data available, women were 27 and males 83. Stage was known for 100 patients: 84 patients had stage I–II disease and 16 had stage III–IV disease. Grade was known for 98 patients: 61 cases were G1–G2 and 37 were G3–G4.

### TMA generation and immunohistochemistry

TMAs were constructed in collaboration with the Unit of Pathology at INT Fondazione Pascale (Naples, Italy) according to established methods [69] using a Tissue Arrayer (Beecher Instruments, Gene Micro-Array Technologies, Silver Spring, MD). Immunostaining was performed using the avidin-biotin-peroxidase method (NovolinKT^M^ Polymer Detection System, Novacastra, Leica Biosystems) on formalin-fixed, paraffin-embedded tissues according to the manufacturer's protocol. Primary antibodies used were: anti-phosho-AKT (Ser473) (Cell Signalling Technology) used at dilution of 1:150 at pH 6, and anti-p27 (BD) used at dilution of 1:1000 at pH 6. The immunohistochemical score of pAKT used in this work was described previously [57]. As for p27, cut-off for scoring tumors as p27-positive or p27-negative was set at 50% [70]. p27 expression was scored nuclear in p27-positive tumors with exclusive nuclear staining and cytoplasmic in p27-positive tumors showing cytoplasmic staining independent of its nuclear positivity.

### Statistical analysis

Data presented are the means ± SD of *n* independent assays or replicates as indicated in the text. Continuous variables were analyzed by Student's *t*-test or ANOVA test, while categorical variables by χ^2^ or Fisher's exact tests. Significance was calculated by Log-rank (Mantel-Cox) test (GraphPad Prizm 5 Software, San Diego, CA).

## SUPPLEMENTARY DATA FIGURES, VIDEOS AND TABLES


