# Supplementary material for: Mutant AKT1-E17K is oncogenic in lung epithelial cells
Source: Oncotarget. 2015 May 25;6(37):39634–50. doi: 10.18632/oncotarget.4022 (PMC4741851; doi:10.18632/oncotarget.4022)
Supplement: Supplementary file 1 [file oncotarget-06-39634-s001.pdf]

SUPPLEMENTARY DATA

AKT1-E17K stimulates proliferation in NSCLC NCI-H23 cells

We confirmed the data obtained in BEAS-2B using NSCLC NCI-H23 cells. Cells were transfected with CMV-based plasmids carrying wild type or mutant AKT1-E17K (H23-pcDNA3, H23-AKT1-WT, H23-AKT1-E17K, respectively). Two clones of H23-PcDNA3, H23-AKT1-WT, H23-AKT1-E17K cells were expanded for biological studies. The presence of the exogenous AKT1-WT or AKT1-E17K proteins in transfected cells was detected by immunoblot (Supplemental Fig. S1A). H23-AKT1-E17K cells presented increased phosphorylation of GSK3α/β (Ser9/22) and FOXO1 (Thr256), particularly in starvation medium. Immunoblot analysis also demonstrated increased membrane localization of mutant AKT1 compared with endogenous or transfected wild type protein as well as increased pS473 phosphorylation of mutant AKT1 (Supplemental Fig. S1B). In addition, immunoblot analysis showed that NCI-H23 cells expressing AKT1-E17K presented decreased amount of nuclear p27 compared with control H23-pcDNA3 or H23-AKT1-WT cells (Supplemental Fig. S1C; 10% vs 40% for p27, respectively).

We also investigated whether AKT1-E17K altered growth potential of NCI-H23 cells. Analysis of cell proliferation by Trypan blue assay demonstrated that H23-AKT1-E17K cells duplicated at an accelerated rate in monolayer compared with H23-pcDNA3 or H23-AKT1-WT cells (Supplemental Fig. S1D). In agreement with the results of AKT activation, the difference in the proliferation rate between H23-AKT1-E17K cells and H23-pcDNA3 or H23-AKT1-WT cells was predominantly observed under conditions of growth factor deprivation.

These results confirm the data obtained in the BEAS-2B cells and indicate that mutant AKT1-E17K, but not wild type AKT1 expressed at similar level, is able to promote anchorage-dependent proliferation of human bronchial epithelial cells, especially under stressful conditions.

AKT1-E17K stimulates migration and invasion in NSCLC NCI-H23 cells

We also evaluated the effects of AKT1-E17K on the capability to migrate of NSCLC cells. All cells were included in 2D Matrigel matrices and followed for 18 hours using time-lapse microscopy. No difference in cell motility was observed in the presence of growth factors (not shown). Conversely, H23-PcDNA3 cells moved with a mean speed of 0.15 ± 0.04 μm/min demonstrating low velocity in serum free conditions. Expression of AKT1-WT did not significantly modify 2D velocity of NCI-H23 cells (0.17 ± 0.04 μm/min). Conversely, AKT1-E17K significantly increased the ability of these cells to move (0.23 ± 0.05 μm/min, $p < 0.0001$) (Supplemental Fig. S2A). The total path described by each cell was significantly longer in AKT1-E17K-expressing cells with respect to control or AKT1-WT expressing cells, demonstrating that AKT1-E17K prevents the reduction of cell speed due to serum deprivation (Supplemental Fig. S2B).

Mutant AKT1 stimulated also anchorage-independent growth in NCI-H23 cells. AKT1-E17K, but not wild type AKT1, induced a significant increase in the number of colonies grown in soft agar compared with parental cells (H23-pcDNA3: 7.3 ± 2.7 colonies/field; H23-AKT1-WT: 9 ± 2.2 colonies/field; H23-AKT1-E17K: 19.3 ± 3.3 colonies/field) (Supplemental Fig. S2C). In addition, when injected subcutaneously into athymic CD1 mice (5×10^6 cells/mouse), mutant AKT1-E17K markedly anticipated tumour formation induced by NCI-H23 cells as illustrated in Supplemental Fig. S2D. Tumors generated by H23-AKT1-E17K cells appeared much earlier (on average at the 4–5th weeks) than those generated by H23-AKT1-WT cells (on average at the 7–8th weeks) or control NCI-H23 cells (10–11th weeks).

These results confirmed the data obtained in the BEAS-2B cells and indicated that mutant AKT1-E17K, but not wild type AKT1 expressed at similar level, is able to stimulate migration, invasion and tumorigenicity in human NSCLC cells.

PIK3CA-E545K and PTEN silencing promote delocalization of p27 in NSCLC cells

Aberrant activation of the PI3K/AKT pathway is a frequent event in NSCLC. It results from gain-of-function mutations of AKT1 itself, of PIK3CA or from PTEN loss. We investigated whether inactivation of p27 by phosphorylation represents a general mechanism whereby abnormally active PI3K/AKT pathway signals in lung epithelial cells. To this aim we generated BEAS-PIK3CA-E545K and BEAS-shPTEN cells after lentiviral-transduction and selection in blasticidin-containing medium (Malanga et al. in preparation). The presence of the exogenous PIK3CA or endogenous PTEN proteins was detected by immunoblot (Malanga et al. in preparation). The status of the PI3K/AKT pathway was determined by analysis of GSK3 phosphorylation (Malanga et al. in preparation). Active PIK3CA (E545K) or PTEN loss are tumorigenic to human lung epithelial cells like mutant AKT1-E17K (Malanga *et al.*, in preparation).

Here we show that, similar to AKT1-E17K, BEAS cells expressing mutant PIK3CA or silenced for PTEN showed a decreased amount of nuclear p27 compared with control BEAS-C cells (Supplemental Fig. S3A) (31% in BEAS-PI3K-E545K, 43% in BEAS-shPTEN versus 58%

of control cells), and higher levels of p27 phosphorylation at T157 and 198, respectively (Supplemental Fig. S3B). Altogether, these results demonstrated that aberrant signalling through the PI3K pathway – induced by mutant AKT1, mutant PIK3CA or by PTEN loss - is associated with T157/T198 phosphorylated p27, which is delocalized in lung epithelial cells. Analysis of cell proliferation by analysis of growth curves indicated that BEAS-PIK3CA and BEAS-shPTEN cells duplicated at an accelerated rate in monolayer compared with BEAS-C (Supplemental Fig. S3C).

SUPPLEMENTAL MATERIALS AND METHODS

Cell culture and transfections

NCI-H23 cells were grown in RPMI supplemented with 10% fetal bovine serum. Transfections were carried out with Fugene 6 reagent (Roche Diagnostics GmbH, Mannheim, Germany). Selection was carried out with 800 mg/ml G418 (Invitrogen, Carlsbad, CA) for 2–3 weeks. Starvation was carried out in RPMI 0.2%.

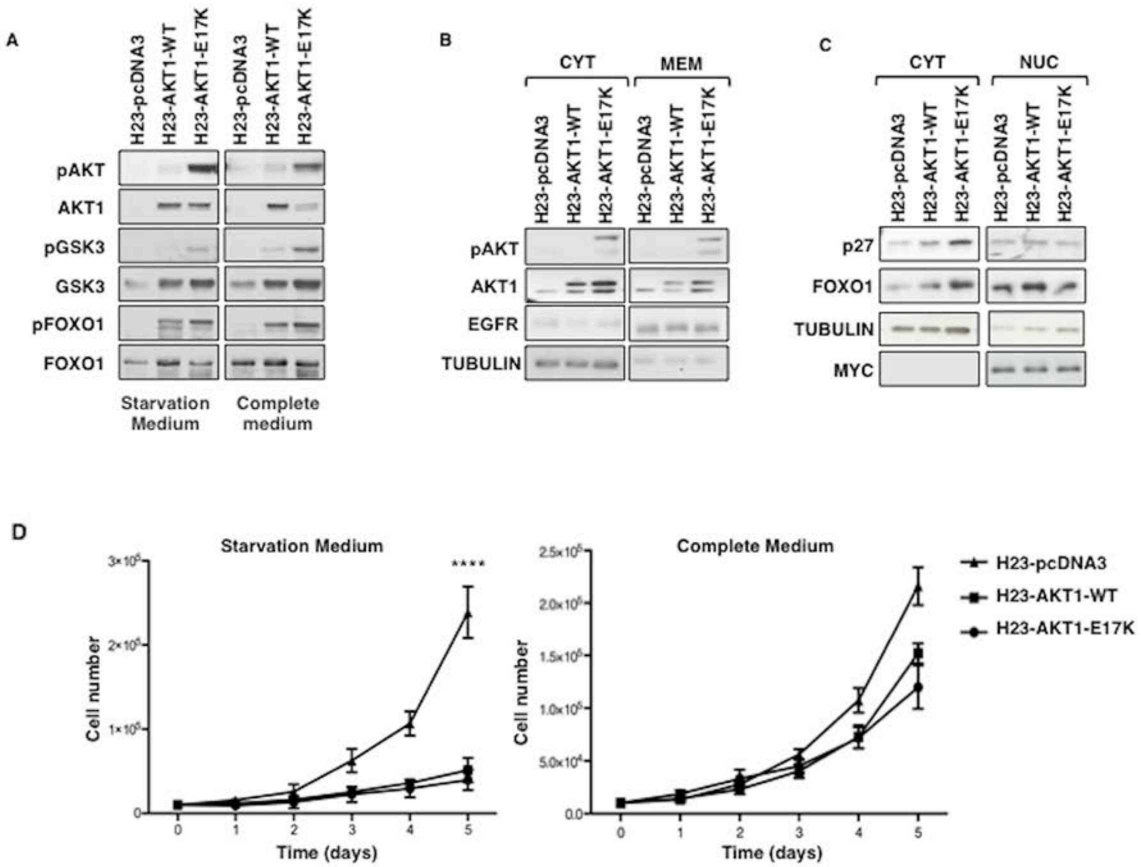

Figure S1: AKT1-E17K increases AKT signalling and cell proliferation rate in NSCLC NCI-H23 cells. **A.** Immunoblot of pAKT, AKT1, pGSK3, GSK3, pFOXO1 and FOXO1 in NCI-H23 cells and derivatives grown in starvation or complete medium. **B.** Immunoblot analysis of pAKT and total AKT1 in extracts enriched in cytosolic or membrane proteins. EGFR and Tubulin were used for normalization and to rule out cross-contamination. **C.** Immunoblot analysis of p27 and FOXO1 in extracts enriched in cytosolic or nuclear proteins. Tubulin and Myc were used for normalization and to rule out cross-contamination. **D.** Trypan Blue exclusion assay of NCI-H23 cells and derivatives. Data are means ± SD from triplicate samples of a representative experiment. ****$p < 0.0001$.

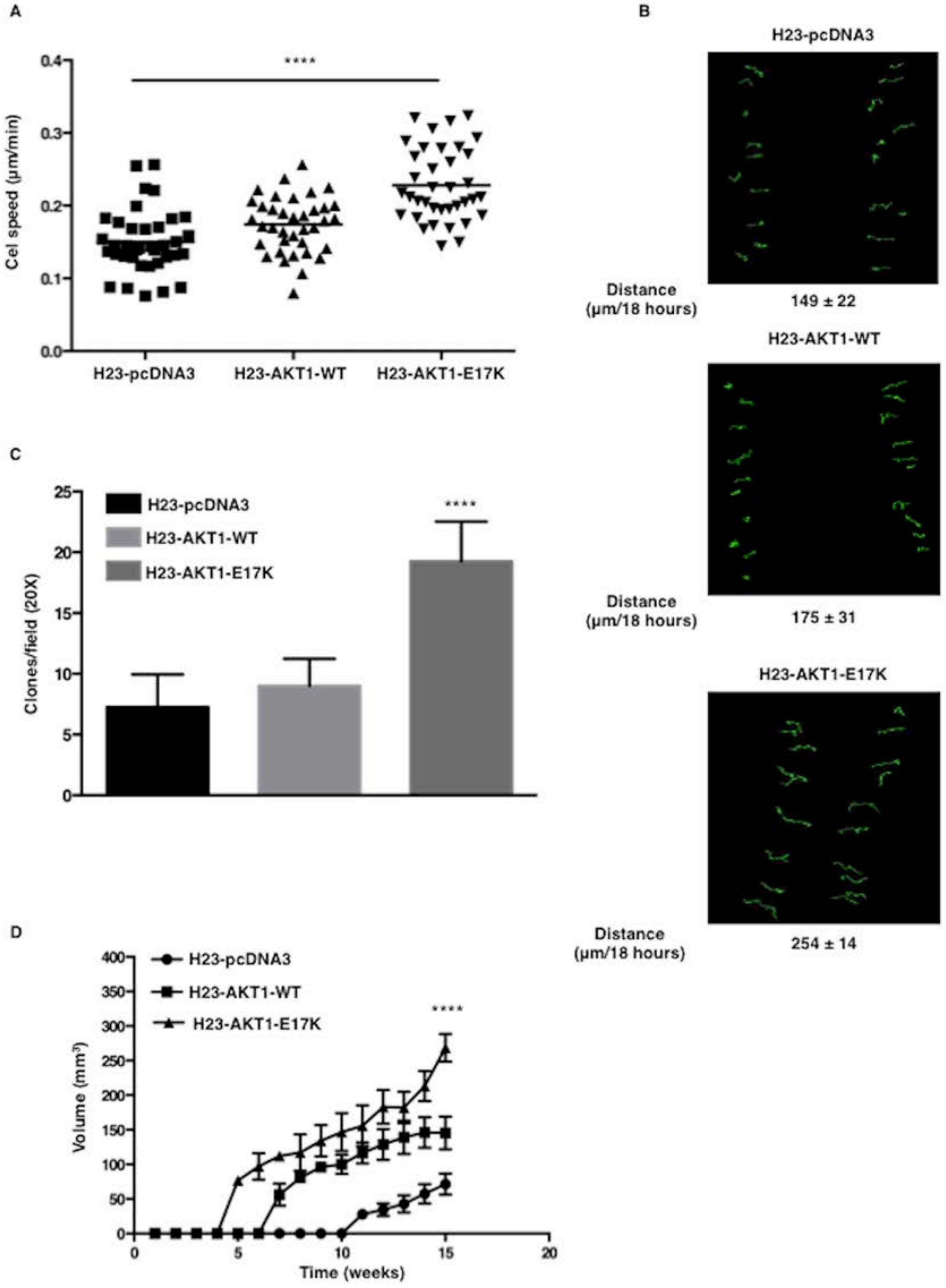

Figure S2: AKT1-E17K promotes motility, invasion and tumorigenesis in NSCLC NCI-H23 cells. A. Cell speed (µµ/min) of NCI-H23 cells and derivatives; bars represent means. ****$p < 0.0001$. **B.** Cell tracking analysis of cells included in 3D Matrigel. In green the trajectories depicted by indicated cell lines. The mean path length (± SD) is reported for each cell line. At least 20 cells were tracked for each cell line in three different experiments. ****$p < 0.0001$. **C.** Soft agar colony formation assay of NCI-H23 and derivatives. Bars represent the mean ± SD of a representative experiment. **D.** NCI-H23 cells and derivatives were injected into the flanks of athymic nude mice ($n = 6$/group). The graph represents the mean (± SD) of tumour volume for each group. ****$p < 0.0001$.

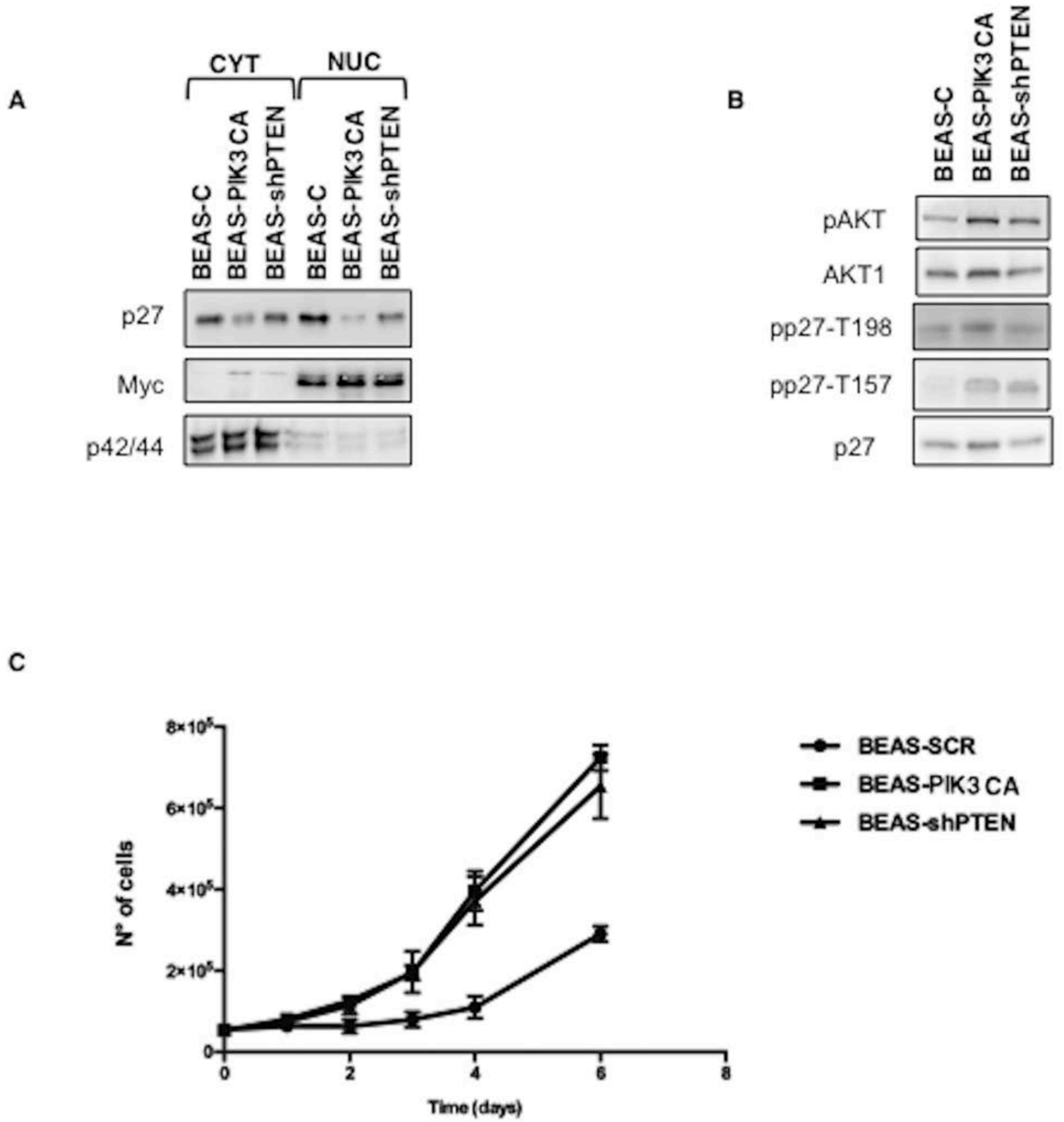

Figure S3: Mutant PIK3CA-E545K allele (BEAS-PIK3CA) and PTEN silencing (BEAS-shPTEN) promote delocalization of cyclin-dependent kinase inhibitor p27 in NSCLC cells. A. Immunoblot analysis of p27 localization in BEAS-C, BEAS-PIK3CA and BEAS-shPTEN cells. **B.** Immunoblot analysis of phospho-198, phospho-T157 and p27 in BEAS-C, BEAS-PIK3CA and BEAS-shPTEN cells. **C.** Trypan Blue exclusion assay of BEAS-C cells and derivatives. Data are means ± SD of triplicate samples of a representative experiment.

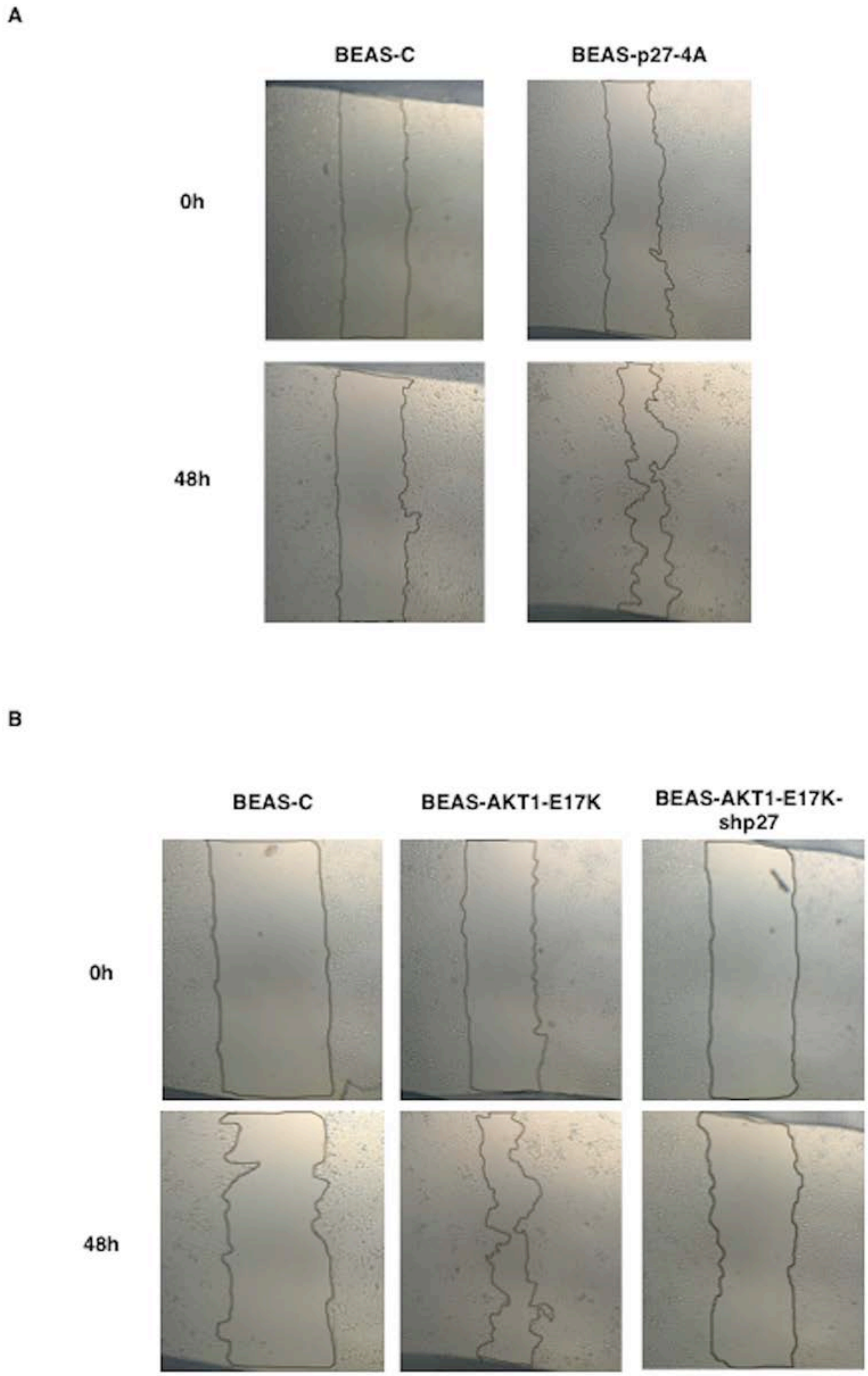

Figure S4: Representative images of wound healing assay. Confluent monolayer cultures of the indicated cells were scratched with a tip and allowed to migrate for 48 hours. **A.** Representative images of BEAS-pcDNA3 and BEAS-p27–4A cells at time 0 (upper panels) and after 48 h (lower panels) from the scratch, respectively. **B.** Representative images of BEAS-C, BEAS-AKT1-WT and BEAS-AKT1-E17K at time 0 (upper panels) and after 48 h (lower panels) from the scratch, respectively. Magnification 4X.

A

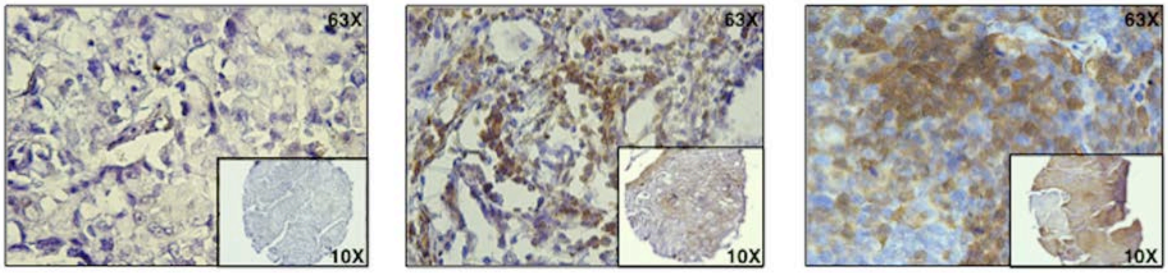

Figure S5: p27 localization in NSCLC. Representative immunostaining of a NSCLC sample showing negative (left panel), nuclear (middle panel) and cytoplasmic (right panel) p27 staining. Magnification as indicated.

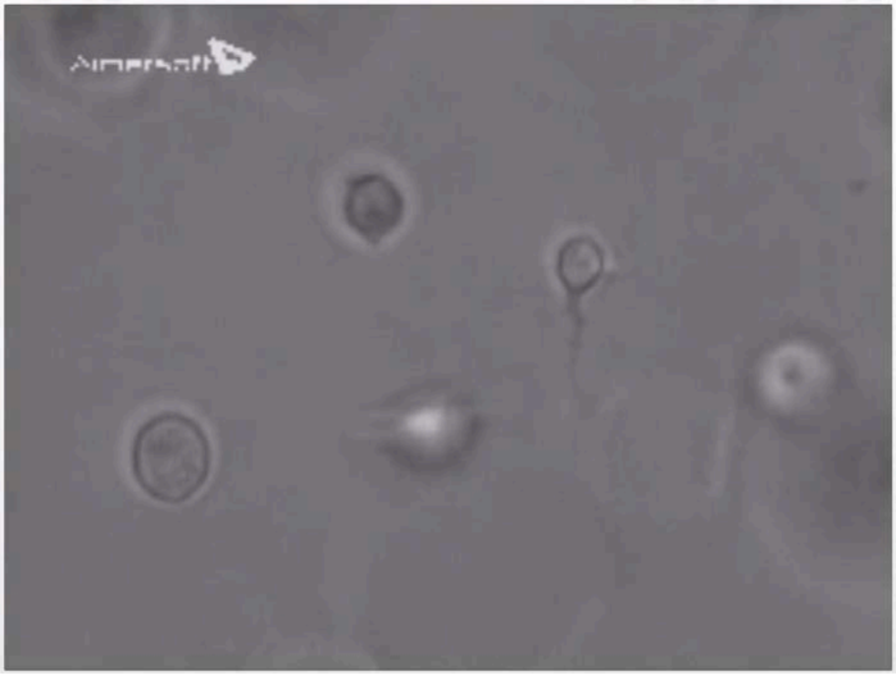

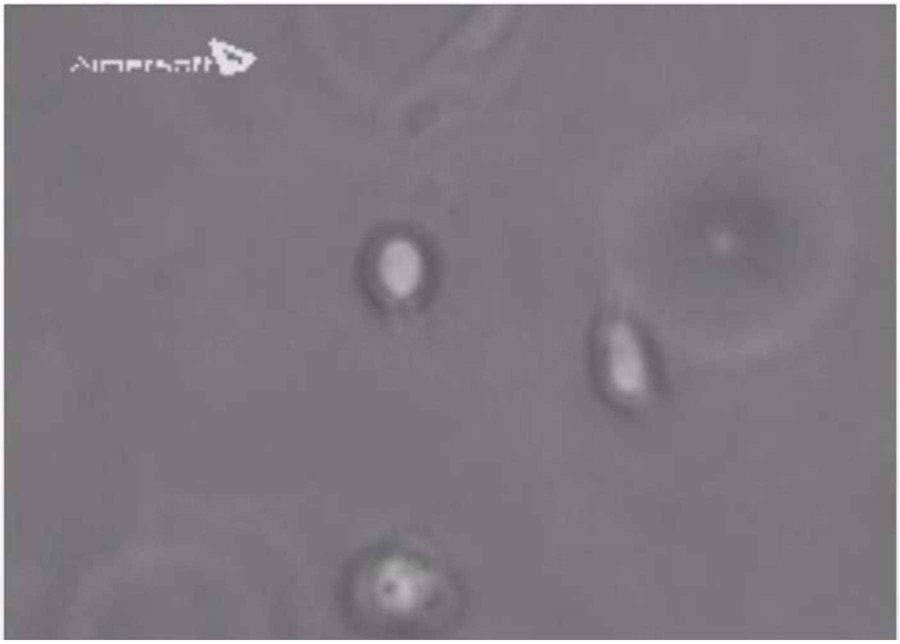

Video S1 and Video S2: BEAS-C and BEAS-AKT1-E17K cells were included in 3D collagen I and their capability to divide and survive was analysed using time-lapse microscopy over a period of 24 hours.

Table S1. Correlation between p27 level/localization and clinico-pathologic features of NSCLC patients

P27

	NEG	POS	NUC	CYT	*P value*
Age[a]					
< 60	3	27	5	22	NS
≥ 60	14	45	13	32	
Grade[b]					
G1–G2	11	36	7	29	NS
G3–G4	6	26	8	18	
Stage[c]					
Ia	7	15	4	11	NS
Ib	3	26	8	18	
IIa	2	6	1	5	
IIb	4	8	1	7	
IIIa-b	1	11	1	10	
Tumour size[d]					
T1	7	23	5	18	NS
T2	8	40	9	31	
T3–T4	2	6	2	4	
NI[e]					
N0	12	54	16	38	NS
N1	4	7	1	6	
Nx	1	9	-	9	

[a] Patients for which p27 staining was available ($N = 89$)
[b] Patients for which both Grade and p27 staining were available ($N = 79$)
[c] Patients for which both Stage and p27 staining were available ($N = 83$)
[d] Patients for which both Tumour size and p27 staining were available ($N = 86$)
[e] Patients for which both Node involvement and p27 staining were available ($N = 87$)
NEG: negative; POS: positive; NUC: nuclear; CYT: cytoplasmic; NS: not significant.

Table S2. Correlation between pAKT staining and p27 level in NSCLC

		p27			
		**Negative**	**Positive**	***N*[a]**	***P value***
**pAKT**	**Negative**	11	35	46	0.157
	**Positive**	4	33	37	
	***N*[a]**	15	68	83	

[a] Patients for which both p27 and pAKT staining was available
